# Altitudinal Effects on Soil Microbial Diversity and Composition in Moso Bamboo Forests of Wuyi Mountain

**DOI:** 10.3390/plants13172471

**Published:** 2024-09-04

**Authors:** Yiming Sun, Xunlong Chen, Jianwei Cai, Yangzhuo Li, Yuhan Zhou, Houxi Zhang, Kehui Zheng

**Affiliations:** 1 College of Forestry, Fujian Agriculture and Forestry University, Fuzhou 350002, China; 5220422032@fafu.edu.cn (Y.S.); 1220496002@fafu.edu.cn (X.C.); tsaijianwei@fafu.edu.cn (J.C.); 3215320195@stu.fafu.edu.cn (Y.L.); 3215303030@stu.fafu.edu.cn (Y.Z.); zhanghouxi@fafu.edu.cn (H.Z.); 2 College of JunCao Science and Ecology, Fujian Agriculture and Forestry University, Fuzhou 350002, China; 3 National Positioning Observation and Research Station of Red Soil Hill Ecosystem in Changting, Fuzhou 350002, China; 4 College of Computer and Information Sciences, Fujian Agriculture and Forestry University, Fuzhou 350002, China

**Keywords:** Moso bamboo forest, altitudes, soil microbial community structure, microbial diversity

## Abstract

Moso bamboo (*Phyllostachys edulis*) forest is a key ecosystem and its soil microbial community plays a crucial role in maintaining the ecosystem’s functions, but it is very vulnerable to climate change. An altitude gradient can positively simulate environmental conditions caused by climate change, and hence, it provides an efficient means of investigating the response of soil microorganisms to such climatic changes. However, while previous research has largely concentrated on plant–soil–microorganism interactions across broad altitudinal ranges encompassing multiple vegetation types, studies examining these interactions within a single ecosystem across small altitudinal gradients remain scarce. This study took Moso bamboo forests at different altitudes in Wuyi Mountain, China, as the research object and used high-throughput sequencing technology to analyze the soil microbial community structure, aiming to elucidate the changes in soil microbial communities along the altitude gradient under the same vegetation type and its main environmental driving factors. This study found that the structure of bacterial community was notably different in Moso bamboo forests’ soil at varying altitudes, unlike the fungal community structure, which showed relatively less variance. Bacteria from *Alphaproteobacteria* phylum were the most dominant (14.71–22.91%), while *Agaricomycetes* was the most dominating fungus across all altitudinal gradients (18.29–30.80%). Fungal diversity was higher at 530 m and 850 m, while bacterial diversity was mainly concentrated at 850 m and 1100 m. Redundancy analysis showed that soil texture (sand and clay content) and available potassium content were the main environmental factors affecting fungal community structure, while clay content, pH, and available potassium content were the main drivers of bacterial community structure. This study demonstrates that the altitude gradient significantly affects the soil microbial community structure of Moso bamboo forest, and there are differences in the responses of different microbial groups to the altitude gradient. Soil properties are important environmental factors that shape microbial communities. The results of this study contribute to a deeper understanding of the impact of altitude gradient on the soil microbial community structure of Moso bamboo forests, thus providing support for sustainable management of Moso bamboo forests under climate change scenarios.

## 1. Introduction

Moso bamboo (*Phyllostachys edulis*) is a rapidly growing, renewable resource that has significant economic and ecological benefits [1,2,3,4]. It is widely distributed in tropical and subtropical areas, especially in China [5,6,7]. Characterizing the factors that control their health and growth is important to better manage Moso bamboo forests in a sustainable way [2,5]. Soil microbials, as key drivers of nutrient cycling and ecosystem functioning, are closely related to plant growth and health [8,9,10]. However, soil microbials are very sensitive to environmental changes [11]. Even subtle environmental shifts can significantly affect their biomass, community structure, and function, and potentially break the balance of nutrient cycling and energy flow within ecosystems [12]. Therefore, understanding the diversity of soil microbial communities in Moso bamboo forests in the context of global climate change is crucial for sustainable management bamboo forests.

In recent years, the intensifying effects of global warming have significantly threatened the stability of terrestrial ecosystems, and the process of soil microbial response to climate warming has become a focal point of research. The IPCC (2021) report indicates that global surface temperature will continue to rise by 3.3–5.7 °C in 2081–2100, which will pose an even more severe test to terrestrial ecosystems [13]. The use of altitudinal gradients to simulate changes in temperature gradients caused by climate change is an important and cost-effective means to study the impact of climate warming on terrestrial ecosystems [14]. Altitudinal gradients significantly influence environmental factors such as temperature, humidity, and light, with shifts in these factors occurring up to 1000 times more rapidly along altitudinal gradients compared to latitudinal ones [15], which means that a more significant climate change gradient can be modeled on a smaller spatial scale. A wide range of environmental factors are influenced by changes in altitude, such as temperature, humidity, and light, which in turn indirectly drive soil microbial metabolism and reproductive processes by regulating forest microclimate, soil physicochemical properties, and vegetation types [14,16]. Therefore, studying the structure of soil microbial community structure characteristics across different altitudinal gradients is crucial for understanding how soil microbes respond to environmental changes, particularly climate warming. This research can provide valuable insights for predicting the potential impacts of future climate change on soil ecological functions.

Currently, studies on the relationship between soil microorganisms and elevation mainly focus on the elevation gradient of ecosystems with large differences or different vegetation types, such as desert grassland, mountain scrub, and different types of forest ecosystems. At the same time, the results of the studies also show diversified trends, such as presenting a rising and then declining trend [17], monotonically declining trend [18], unimodal change trend [19], etc., and have not yet formed a consistent conclusion. The reasons for such differences may be related to a variety of factors such as climatic conditions, soil types, vegetation composition, and research methods in the study area. However, there is a lack of research on the changing law of soil microorganisms along the altitudinal gradient in a single ecosystem or the same vegetation type, and this aspect of research is of great significance for a deeper understanding of the microbial mechanism of nutrient cycling processes in forest ecosystems, as well as the role of microbial communities in maintaining the stability of these ecosystems [14].

Wuyi Mountain, located in the southeast region of China, has the most extensive and well-preserved meso-subtropical forest ecosystem at the same latitude in the world, and its natural altitude difference of 1900 m forms a complete vertical zone spectrum of vegetation. Moso bamboo forests, distributed across various altitudes, provide an ideal setting for investigating the relationship between soil microbial communities and altitudinal gradients within these ecosystems.

Therefore, in this study, we analyzed the soil microbial diversity and community structure in the Moso bamboo forests across different altitudes in Wuyi Mountain, with a view to elucidating the patterns of change in soil microbial communities along the different altitudes of the same vegetation type and the primary driving factors. The findings of this study may offer a theoretical basis for understanding the change patterns of the microbial communities in the context of global warming in the future, and provide scientific support for the sustainable management of the ecosystems of the Moso bamboo forests ecosystems.

## 2. Materials and Methods

### 2.1. Study Area

**Figure 1:**
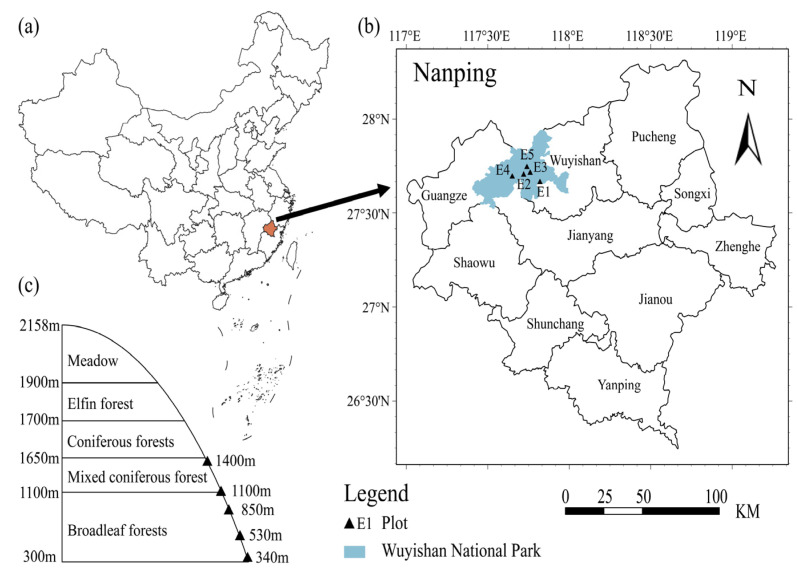
Locations of the study area (**a**,**b**) and the sampling sites of Moso bamboo forests along the different altitudes in Wuyi Mountain (**c**).

Wuyi Mountain, located at the border between the northwest of Fujian Province and Jiangxi Province, is characterized by a subtropical monsoon climate (Figure 1a). The area experiences hot and rainy summers and mild, rainy winters. The average annual temperature is 18.3 °C, with an annual average precipitation of about 2000 mm. The mountain also averages over 100 foggy days per year, with an average relative humidity of 83.5%. The region features complex terrain and a rich diversity of geomorphological types and vegetation [20,21]. From the foot to the summit of the mountain, there is a significant variation in hydrothermal conditions; every 100 m increase in elevation results in a temperature decrease of 0.44 °C and a precipitation increase of 37.00 to 54.14 mm [20,21], leading to a distinct vertical zonation of vegetation and soil. Moso bamboo has a long history of cultivation on Wuyi Mountain, with a broad planting area distributed across various elevations ranging from 250 to 1500 m [20,21].

### 2.2. Sample Setting and Soil Sampling

The Moso bamboo forest is an important vegetation type in the study area, which is distributed in a wide altitudinal range (250–1500 m). The management practices of Moso bamboo forests are extremely rough, with little use of fertilizer management measures, primarily focusing on timber production. From May to June 2016, we established sampling plots of the Moso bamboo forests along specific altitudinal gradients in the Wuyi Mountain Nature Reserve according to the references. In July 2016, soil samples were collected from Moso bamboo forests on Wuyi Mountain. The elevation range of 250–1500 m was divided into five distance gradients at regular intervals (Figure 1b). Within each elevation gradient, three pure Moso bamboo forest sample plots (projected area 10 m × 10 m) were selected, and the latitude and longitude of each plot were recorded using a handheld GPS (Magellan Explorist 610) (Figure 1b,c). Within each plot, three randomly designated soil sampling areas were established, with five soil sampling points arranged in an “S” shaped in each area. After removing the surface layer of litter, topsoil samples (0–10 cm) were collected using a sterile soil auger. Soil from the same plot was mixed homogeneously and then placed in sterile zipper bags and stored in portable ice boxes. At the same time, soil at the sampling site was sampled using a 5 cm diameter ring cutter, which was used to determine the soil bulk weight. The soil samples obtained from the soil auger were mixed again in a sterile environment. After being passed through a 2 mm sieve, the sample was divided into two parts. One portion was used for high-throughput sequencing and stored at −80 °C in an ultra-low-temperature refrigerator, while the other portion was air-dried before further analysis of soil physicochemical properties. Note: The data on soil organic carbon (SOC) and available potassium (AK) were sourced from Zhang Houxi et al. [22]. (See Figure 1 and Table 1).

### 2.3. Soil Physical and Chemical Properties Determination

The soil physicochemical properties measured included soil pH, bulk density, organic carbon, total nitrogen (TN), total phosphorus (TP), available phosphorus (AP), total potassium (TK), and available potassium [23]. Soil pH was determined using a potentiometric method with a soil-to-water ratio of 2.5:1. Bulk density was measured using the ring knife method. Soil organic carbon was quantified using the potassium dichromate external heating method. Total nitrogen was determined by the Kjeldahl method. Total phosphorus was measured using the hydrofluoric acid–perchloric acid digestion followed by the molybdenum–antimony colorimetric method. Available phosphorus was extracted with sodium bicarbonate and measured using the molybdenum–antimony colorimetric method. Total potassium was determined by hydrofluoric acid–perchloric acid digestion followed by flame photometry. Available potassium was extracted with ammonium acetate and measured using flame photometry.

### 2.4. DNA Extraction and High-Throughput Sequencing

Total DNA was extracted from soil samples (0.5 g) using the HiPure Soil DNA kit (Magen, Guangzhou, China) according to the operating instructions. The concentration and quality of DNA were assessed by a NanoDrop 2000 spectrophotometer (ThermoScientific, Wilmington, DE, USA). PCR amplification of bacterial 16S rRNA targeting the V3–V4 region was performed using primers 341F (CCTACGGGGNGGCWGCAG), 806R (GGACTACHVGGGGTATCTAAT) [24]; PCR amplification of bacterial 16S rRNA targeting the V3-V4 region was performed using primers CTTGGTCATTTAGAGAGGAAGTAA), and ITS2R (GCTGCGTTCTTCATCGATGC) to achieve amplification of the fungal ITS1 region [25]. PCR reactions were performed in triplicate with 30 cycles of 95 °C for 5 min, then 95 °C for 1 min, 60 °C for 1 min, and 72 °C for 1 min, and a final cycle of 72 °C for 7 min. Amplicons were collected from 2% agarose gels using the AxyPrep DNA Gel Extraction Kit (Axygen Biosciences, Union City, CA, USA) for purification according to the instructions and quantified using the ABI StepOnePlus Real-Time PCR System (Life Technologies, Foster City, CA, USA). The purified amplicons were subjected to double-end sequencing (PE250) on the Illumina platform according to standard practices [26,27]. 

### 2.5. Statistical Analysis

Data statistical analysis was conducted using SPSS software version 22.0. Differences in soil physicochemical properties were compared using one-way Analysis of Variance (ANOVA), with significant differences determined by the Least Significant Difference (LSD) multiple comparison test (*p* < 0.05). Stacked charts of fungal and bacterial species distribution as well as bar charts of alpha diversity indices were created using Excel 2019. Data statistical analysis was conducted using SPSS software version 22.0. Differences in soil physicochemical properties were compared using one-way Analysis of Variance (ANOVA), with significant differences determined by the Least Significant Difference (LSD) multiple comparison test (*p* < 0.05). Stacked charts of fungal and bacterial species distribution as well as bar charts of alpha diversity indices were created using Excel 2019. Venn diagrams was generated using the VennDiagram package in the R (R-4.3.2). Alpha diversity indices including ACE, Chao, and Shannon indices were calculated and the Shannon indices of the samples were tabulated; in order to explore the similarity of microbial community structure among the samples, Principal Coordinates Analysis based on the Bray-Curtis distance algorithm was applied. Redundancy Analysis (RDA) ordination was performed with CANOCO 5.0, which further used the forward screening method and the Monte Carlo test to quantify the contribution of different soil physicochemical properties and two-dimensional ordination plots were produced to visualize the data.

## 3. Results

### 3.1. Structure of Soil Microbial Communities in Moso Bamboo Forests at Different Altitudes

#### 3.1.1. OTU Cluster Analysis

**Figure 2:**
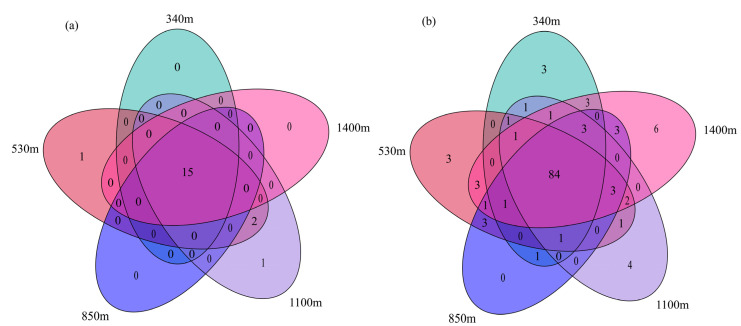
Venn diagram of soil fungi (**a**) and bacteria (**b**) in Moso bamboo forests at different altitudes in Wuyi Mountains.

The Venn diagram clearly reflects the number of shared and unique OTUs among the samples. As shown in Figure 2, there are 15 shared fungal OTUs and 84 shared bacterial OTUs across the samples. Soil samples from elevations adjacent to the mid-elevation (530 m and 1100 m) contained one unique fungal OTU, while soils from the other three elevations had no unique fungal OTUs. Low elevation sites (340 m and 530 m) harbored three unique bacterial OTUs, while high elevation sites (1100 m and 1400 m) had four and six unique bacterial OTUs, respectively. In contrast, the mid-elevation site (850 m) had no unique bacterial OTUs. This suggests that the composition of the bacterial community varies considerably at different altitudes, whereas differences in the fungal community are less pronounced.

#### 3.1.2. Fungal Community Structure

**Figure 3:**
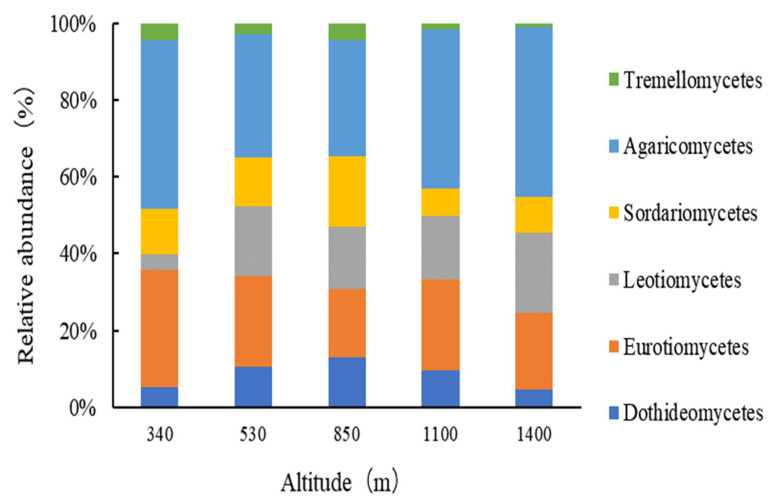
Relative abundances of the dominant fungal classes in Moso bamboo forests along the elevational gradient.

Fungi were classified into six classes (excluding those with a relative abundance of less than 2%, as shown in Figure 3): *Agaricomycetes*, *Eurotiomycetes*, *Leotiomycetes*, *Sordariomycetes*, *Dothideomycetes*, and *Tremellomycetes*. Among these, *Agaricomycetes*, *Eurotiomycetes*, and *Leotiomycetes* were the dominant fungal classes in the soils of Moso bamboo forests across different elevations of Wuyi Mountain, with relative abundances ranging from 18.29% to 30.80%, 10.79% to 16.76%, and 2.20% to 14.52%, respectively. At different elevations, *Agaricomycetes* consistently exhibited the highest relative abundance. At the highest elevation (1400 m), the relative abundance of *Eurotiomycetes* was less than that of *Leotiomycetes*, whereas at the other four elevations, *Eurotiomycetes* was more abundant than *Leotiomycetes*.

**Figure 4:**
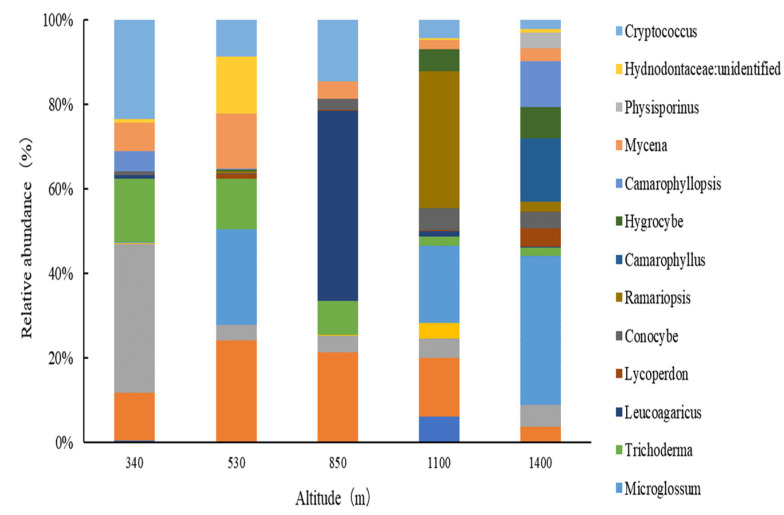
Relative abundances of the dominant fungal groups in Moso bamboo forests along the elevational gradient.

At the genus level, seventeen fungal groups were identified (excluding those with a relative abundance of less than 2%, as shown in Figure 4), and major fungal genera in soils at different elevations differ considerably. At lower elevations (340 m and 530 m), the dominant genera were *Oidiodendron*, with relative abundances of 1.11–4.4%, *Microglossum*, ranging from 0.02% to 4.11%, and an unidentified genus from the family *Herpotrichiellaceae*, with abundances of 0.66–3.39%. At mid-elevation (850 m), the dominant genera were *Leucoagaricus*, with a relative abundance of 7.45%, *Oidiodendron*, at 3.52%, and *Cryptococcus*, at 2.42%. At higher elevations (1100 m and 1400 m), the dominant genera were *Microglossum*, ranging from 4.46% to 8.51%, Ramariopsis, between 0.55% and 7.93%, and *Oidiodendron*, from 0.85% to 3.44%.

#### 3.1.3. Bacterial Community Structure

**Figure 5:**
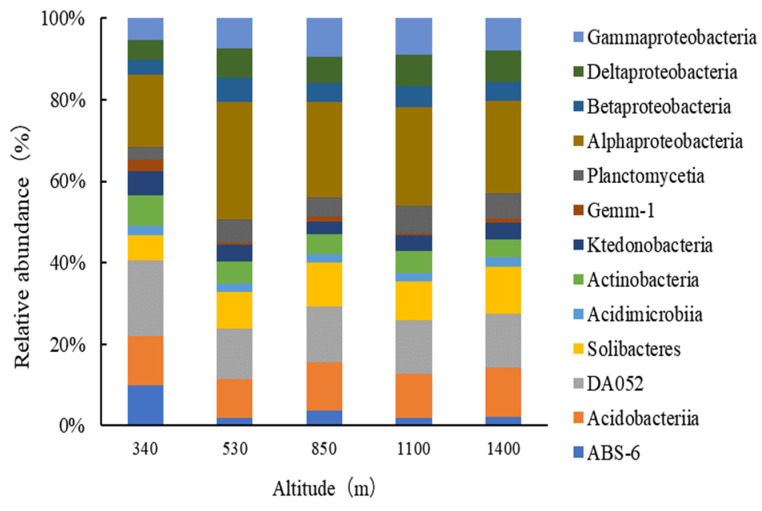
Relative abundances of the dominant bacterial classes in Moso bamboo forests along the elevational gradient.

Bacteria were classified into 13 classes (excluding those with a relative abundance of less than 2%, as shown in Figure 5). *Alphaproteobacteria* was the most prevalent, with a relative abundance ranging from 14.71% to 22.91%. Other significant classes included *DA052*, *Acidobacteriia* and *Solibacteres*, with relative abundances of 9.80–15.38%, 7.65–10.00%, and 5.25–8.98%, respectively. *Acidobacteriia* and *DA052* were most abundant at the lowest elevation (340 m), at 10.00% and 15.38%, respectively, and least abundant at a lower elevation (530 m), at 7.65% and 9.80%, respectively. *Alphaproteobacteria* showed the lowest abundance at the lowest elevation (14.71%) and the highest at a lower elevation (22.91%). *Solibacteres* reached its highest abundance at the highest elevation (1400 m), and its lowest at the lowest elevation.

**Figure 6:**
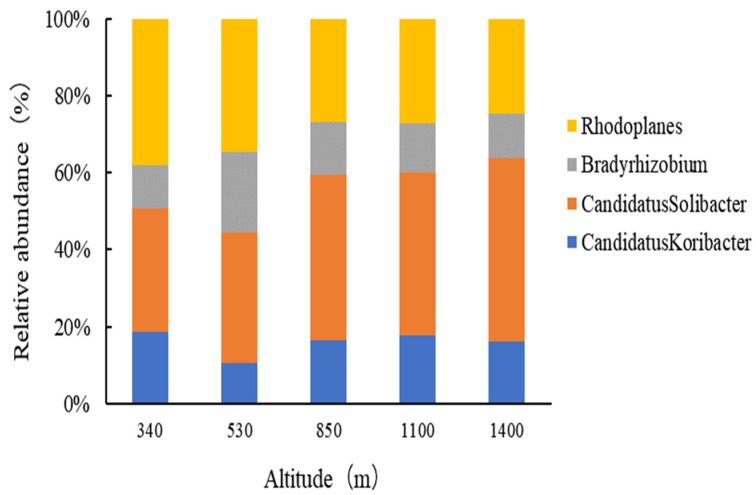
Relative abundances of the dominant bacterial groups in Moso bamboo forests along the elevational gradient.

At the genus level, four bacterial groups were identified (excluding those with a relative abundance of less than 2%, as shown in Figure 6), among which *Candidatus Solibacter* and *Rhodoplanes* were the most prevalent. In soils at lower elevations (340 m and 530 m), *Rhodoplanes* constituted 4.30% and 5.05%, respectively, higher than *Candidatus Solibacter*, which constituted 3.64% and 4.93%. However, in mid-elevation (850 m) and higher elevation soil samples, the average content of *Candidatus Solibacter* (6.03%) was higher than that of *Rhodoplanes* (3.57%).

#### 3.1.4. Diversity Indices

**Figure 7:**
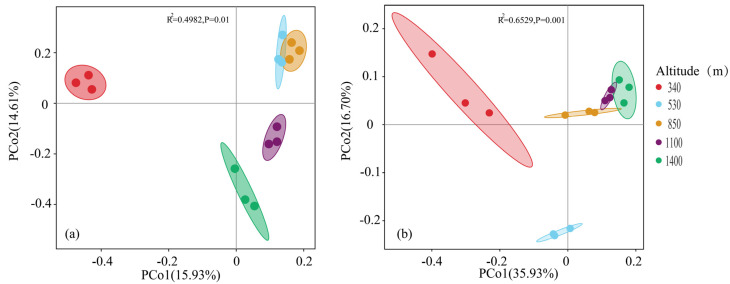
Principal coordinates analysis of soil fungi (**a**) and bacteria (**b**) community structure in Moso bamboo forests at different elevations in Wuyi Mountain.

Alpha diversity describes the species diversity within a specific region or ecosystem and can comprehensively reflect the richness and evenness of the microbial community [28,29]. This study used CHAO and ACE indices to assess the diversity and richness of microbial species. As shown in Table 2, the trends of fungal CHAO and ACE are consistent, and the fungal CHAO and ACE indices at altitudes of 530 m and 1100 m are higher than those at 340 m and 14,000 m altitudes (*p* < 0.05). However, the results showed that the fungal Shannon index showed a trend of first increasing and then decreasing, with significant differences between the Shannon index at 530 m, 850 m, and 1400 m (*p* < 0.05). The bacterial Shannon index did not show an obvious trend along the altitude gradient and was only significantly different from other altitudes at 340 m (*p* < 0.05). In summary, this study found that fungal diversity was higher at 530 m, 850 m, and 1100 m above sea level, while bacterial diversity was mainly concentrated at 850 m and 1100 m. To further explore the differences in soil microbial community structure in Moso bamboo forests at different altitudes in Wuyi Mountain, this study conducted a differential analysis of soil microbial community structure, i.e., a Beta diversity assessment. This study used the Principal Coordinates Analysi (PCoA) to analyze the differences in species composition between different regions. The results showed that the first two ranking axes of PCoA explained 52.63% and 30.54% of the variation in bacterial and fungal samples, respectively (Figure 7), which implies that there were obvious differences between the soil bacterial and fungal communities in Moso bamboo forests at different altitudes. In addition, the bacterial aggregation levels at 340 m and 530 m are close, while the fungal community aggregation levels are close at 1100 m and 1400 m, indicating that the composition and structure of bacteria and fungi at these two altitudes are relatively similar.

### 3.2. Relationship between Soil Microbial Community Structure and Environmental Factors

To elucidate the intricate relationship between soil microorganisms (fungi and bacteria) and environmental factors (Table 1), this study used the redundancy analysis (RDA). This multivariate statistical method allowed us to assess the influence of selected environmental variables on the composition of soil microbial communities. 

**Figure 8:**
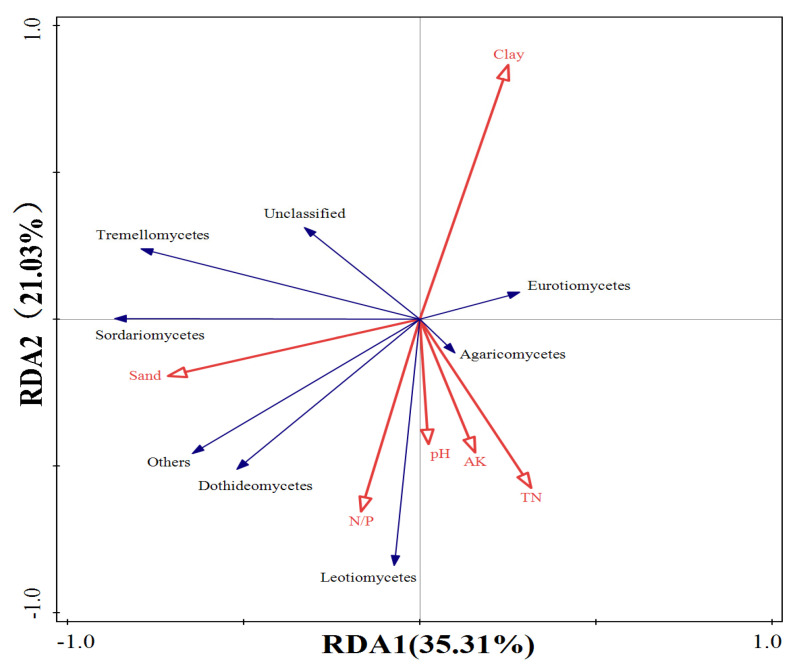
Redundant analysis of fungal community structure and environmental factors.

The RDA results for fungi showed that the first two ordination axes explained 35.31% and 21.03% of the variation in soil fungal community structure, respectively (Figure 8), accounting for a total of 56.34%. This indicates that the selected environmental factors can explain most of the variation in bacterial community structure. Among them, clay particles were positively correlated with the abundance of *Eurotiomycetes* while negatively correlated with other fungal communities. Sand particles negatively correlated with the abundance of *Eurotiomycetes* and *Agaricomycetes* but positively correlated with other fungal communities. The N/P ratio was negatively correlated with *Eurotiomycetes* and *Tremellomycetes* but positively correlated with other fugal communities. Soil pH negatively correlated with *Eurotiomycetes*, *Tremellomycetes*, and *Sordariomycetes* while positively correlated with other communities. Available potassium and total nitrogen negatively correlated with *Sordariomycetes* and *Tremellomycetes* but positively correlated with other fungal communities.

**Figure 9:**
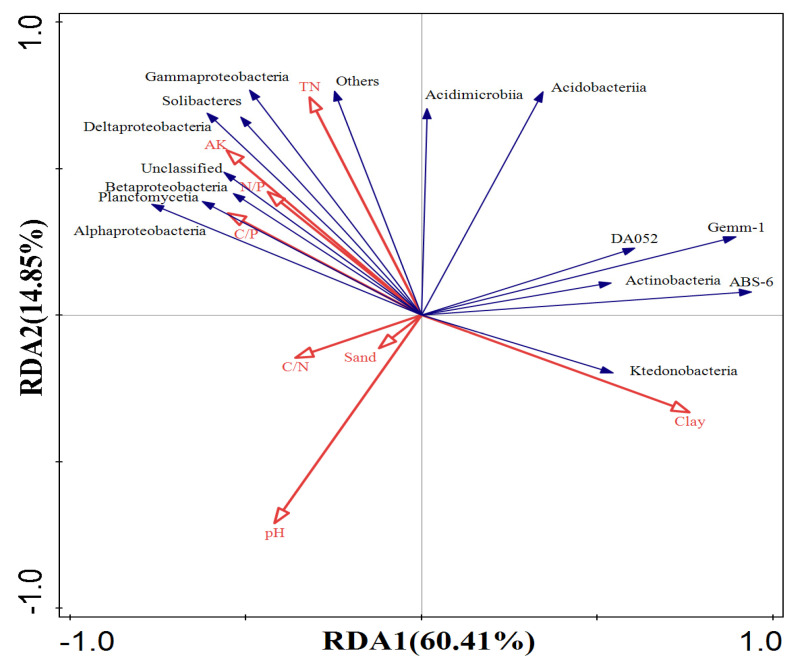
Redundant analysis of bacterial community structure and environmental factors.

The RDA results for bacteria demonstrated that the first two ordination axes explained 60.41% and 14.85% of the variation in soil bacterial community structure, respectively (Figure 9), accounting for a total of 75.26%. This suggests that the selected environmental factors can explain most of the variation in bacterial community structure. Among them, pH and sand particles were negatively correlated with the majority of bacterial communities. The C/N ratio showed a balanced influence, with an almost equal number of communities exhibiting positive and negative correlations. C/P, N/P, available potassium, and total nitrogen were negatively correlated with specific bacterial groups (*DA052*, *Gemm-1*, *ABS-6*, *Actinobacteria*, and *Ktedonobacteria*), while positively correlated with other communities. Clay particles were positively correlated with *DA052*, *Gemm-1*, *ABS-6*, *Actinobacteria*, and *Ktedonobacteria*, but negatively correlated with other communities.

While RDA effectively visualizes the relationship between microbial communities and soil physical and chemical properties, quantifying the contribution of different factors to soil microbials still poses a challenge. Therefore, we further used the forward screening method and Monte Carlo test to quantify the contribution of each environmental factor. As shown in Table 3, the explanatory power of environmental factors on the variation of fungal structure followed the order: sand (19.6%), clay (17.3%), available potassium (15.9%), pH (4.7%), N/P (2.3%), and total nitrogen (1.7%). Among them, sand, clay, and available potassium have significant effects on the variation of fungal community structure (*p* < 0.05) (Table 3). The explanatory power of environmental factors on the variation of bacterial community structure was ranked as follows (Table 4): clay (37.3%), pH (17.1%), available potassium (10.3%), N/P (6.4%), total nitrogen (4.8%), C/P (3.9%), sand (2.8%), and C/N (1.3%). Similar to fungi, pH, clay, and available potassium have significant effects on the variation of bacterial community structure (*p* < 0.05) (Table 4).

## 4. Discussion

### 4.1. Characterization of Soil Microbial Community Structure in Moso Bamboo Forests

This study shows that the soil fungi in the bamboo forest of Wuyi Mountain mainly include *Agaricomycetes*, *Eurotiomycetes*, *Leotiomycetes*, *Sordariomycetes*, *Dothideomycetes*, and *Tremellomycetes*, which is similar to the composition of many other ecosystems [30,31], including similar compositions found in vegetation ecosystems in semi-arid areas [32]. Studies have shown that fungal communities play a crucial role in improving the soil environment and are of great significance for maintaining the stability of forest ecosystems [33]. This study found that the dominant fungi in the bamboo forest soil at different altitudes in Wuyi Mountain are *Agaricomycetes* (18.29–30.80%), *Eurotiomycetes* (10.79–16.76%), and *Leotiomycetes* (2.20–14.52%). Among them, *Agaricomycetes* belonging to the phylum Basidiomycetes have a strong ability to decompose wood components and can decompose the difficult-to-decompose lignin and cellulose in litter to enhance the physical properties of the soil. They also play an important role in nutrient cycling, such as degrading soil organic matter and promoting soil carbon accumulation. At the same time, their mycelium has a strong ability to spread, and their spores can complete long-distance diffusion and the establishment of primary bacterial communities [34,35], thus becoming the dominant bacterial community in soil fungi; secondly, *Leotiomycetes* fungi belonging to the phylum Ascomycetes have strong adaptability, can survive in extreme environments, and are widely distributed around the world [36,37]. Other studies have shown that Ascomycota and Basidiomycota are the main fungal communities involved in soil organic matter decomposition, and are closely related to the plant rhizosphere nitrogen cycle, plant mycorrhizal formation, and plant growth and development. This shows that the enrichment of Ascomycota and Basidiomycota is conducive to the growth and development of bamboo [38]. Therefore, the rich fungal communities in the bamboo forest soils at different altitudes in Wuyi Mountain are of great significance for maintaining the healthy and sustainable development of the local bamboo forest ecosystem. This study also found that the proportion of *Agaricomycetes* in the soil at medium altitude was the lowest among the five altitude soils, while some studies have found that the proportion of *Agaricomycetes* decreased by 19.9% after planting vegetation [39]. The vegetation diversity in the altitude area of this study was high. We speculate that the rhizosphere secretions of some vegetation may have an inhibitory effect on the survival of *Agaricomycetes*.

This study indicated that the main constituents of the bacterial community in Moso bamboo forest soils in Wuyi Mountain were *Alphaproteobacteria*, *DA052*, *Acidobacteriia*, and *Solibacteres*, with *Alphaproteobacteria* as the most dominant. This might be related to the fact that Proteobacteria have become very adaptable and hence are able to grow and exist in most environments [40]. In our study, high proportions of acidobacterial groups *DA052* and *Acidobacteriia* might be due to the acidic soil conditions (pH 4.21–4.96), which commonly prevails in Wuyi Mountains. This is suitable for some acidobacteria [41]. A negative correlation between *Alphaproteobacteria* and *Acidobacteriia* was also observed at the class level. This is in agreement with their strategy for nutrition, as *Alphaproteobacteria* are copiotrophic and therefore require much nutrition from the soil, while *Acidobacteriia*, as oligotrophs, experience growth suppression with increasing nutrient availability [42].

### 4.2. Factors Affecting Soil Microbial Communities in Moso Bamboo Forests

This study observed the highest fungal diversity index in the mid-altitude Moso bamboo forest soils. However, previous studies have reported a decreasing trend or no significant changes in fungal community diversity with increasing altitude [43]. There may be many reasons for this inconsistency. Firstly, the mid-altitude regions in Wuyi Mountains have favorable climatic conditions and rich vegetation diversity, and plant root exudates will significantly influence fungal communities [44]. Secondly, some studies suggested that within a certain altitudinal range, fungal communities remain unaffected by altitude [44], and soil physicochemical properties, such as organic carbon, total nitrogen, available phosphorus, available potassium, pH, and electrical conductivity, outweigh the influence of altitude itself on fungal communities [41,43,45].

In contrast to fungal diversity, we did not find any significant altitudinal pattern for bacterial Shannon diversity index in Moso bamboo forest soils. This contradicts numerous studies that have reported a monotonic decrease in bacterial diversity with increasing altitude [46]. This discrepancy could be derived from the indirect influence of altitude on microbial community distribution by altering environmental factors such as temperature and humidity, as supported by studies on bacterial communities in the Changbai Mountains [47]. Moreover, the patterns of bacterial diversity and composition patterns have a close relationship with soil pH, just as has been shown by research conducted in the Baetic Cordillera, where altitude Itself did not show a significant direct impact on soil bacterial communities but indirectly affected them by altering plant community composition [48].

Our study found that pH was one of the major factors influencing bacterial communities, consistent with the results reported in many studies [49,50]. However, we found no significant effect of pH on fungal communities, which may be due to the low abundance of fungal taxa that are sensitive to pH changes in our soil samples, which is similar to the findings of Johannes et al. [51]. Another major soil property that affects the structure of soil microbial communities in bamboo forests is soil particle size. Our results indicated a significant impact of clay content on bacterial community structure, while both sand and clay content significantly affected fungal communities. This is in line with previous studies that showed that soil particle size had a great effect on microbial community diversity indices [52,53]. However, some studies have reported no significant impact of soil particle size on microbial community composition [54]. This difference may be caused by variations in climatic conditions across study regions. For example, in mid-latitude regions, soil particle size significantly influences soil water holding capacity, which indirectly affects microbial activity. In contrast, our study site, situated in a subtropical region with high soil moisture, experiences substantial fluctuations in soil water content due to even slight changes in soil particle size, ultimately impacting microbial activity.

Furthermore, our study revealed that the available potassium content in the soil had a significant effect on both bacterial and fungal communities. This is consistent with the results of many studies. For example, in a study of Chinese fir forests, it was found that the available potassium content was significantly positively correlated with soil microbial biomass [50]; Wang et al. [55] found in their study of the forest ecosystem on the Qinghai-Tibet Plateau that available potassium had an important effect on soil microorganisms, second only to soil moisture. This may be because the available potassium content in the soil of the sample site in this study was high, and soil microorganisms mainly existed in large pores or on the surface of aggregates. Consequently, soil microenvironment nutrients will directly affect microbial growth and reproduction, ultimately affecting the community structure and distribution of microorganisms [56].

In summary, our study provides preliminary insights into the altitudinal patterns of soil microbial communities within a specific range in Moso bamboo forests. Further in-depth research is required in the future to reveal the key driving factors and mechanisms responsible for these observed patterns in Moso bamboo forests in Wuyi Mountain.

## 5. Conclusions

This study highlights the significant influence of altitudinal gradients on soil microbial community structure and diversity in Moso bamboo forests of Wuyi Mountains. Clear differences in the fungal and bacterial community structures were found according to the different altitudinal gradients, together with changes in the dominant taxon composition. Noticeably, the highest diversity for fungi was found at middle altitudes, while that of bacteria was at higher altitudes, therefore suggesting differential responses by microbial groups to altitudinal gradients. More precisely, the study evidenced different drivers of the structure of the microbial community among different microbial groups and especially soil physiochemical properties. These findings could provide insight about altitudinal distribution and driving mechanisms shaping soil microbial communities in Moso bamboo ecosystems. In addition, they provide the baseline of possible shifts in soil microbial communities under scenarios of future climatic change.

## Figures and Tables

**Table 1:** Basic information of the sampled plots. Different letters indicate significant differences (*p* < 0.05).

ID	Altitude (m)	Longitude (°)	Latitude (°)	SOC (g/kg)	TN (g/kg)	TP (g/kg)	AP (mg/kg)	AK (mg/kg)	C/N	C/P	N/P	BD (g/cm^3^)	pH
E1	340	117.82	27.67	25.69 b	2.65 b	0.35 b	3.75 c	49.72 b	17.11 a	126.04 c	7.43 d	0.98 a	4.47 a
E2	530	117.72	27.71	34.41 b	2.86 b	0.24 b	2.45 c	63.89 b	22.47 a	256.41 ab	12.41 bc	1.07 a	4.96 c
E3	850	117.76	27.72	54.87 a	5.42 a	0.34 b	7.09 b	75.28 b	18.15 a	291.48 ab	16.06 ab	0.93 a	4.21 d
E4	1100	117.65	27.70	59.20 a	6.12 a	0.34 b	7.69 b	106.11 a	16.92 a	305.36 a	18.22 a	0.63 b	4.80 bc
E5	1400	117.74	27.75	70.68 a	6.10 a	0.71 a	11.72 a	117.22 a	20.04 a	173.53 bc	8.61 cd	0.70 b	4.42 b

**Table 2:** The alpha diversity analysis of soil fungal and bacterial communities in Moso bamboo forests at different elevations in Wuyi Mountain. Different letters indicate significant differences (*p* < 0.05).

	Altitude (m)	CHAO	ACE	Shannon
	340	2273.91 ± 315.38 a	2126.98 ± 205.77 a	7.09 ± 0.19 ab
	530	3316.08 ± 237.74 b	3134.75 ± 193.43 b	7.86 ± 0.35 a
Fungi	850	2627.20 ± 393.18 ab	2575.24 ± 278.29 ab	7.96 ± 0.31 a
	1100	3278.24 ± 438.06 b	3046.39 ± 401.11 b	7.50 ± 0.52 ab
	1400	2010.11 ± 804.18 a	1907.70 ± 711.76 a	6.86 ± 0.91 b
	340	14,174.60 ± 1794.40 a	14,561.00 ± 1914.33 a	9.83 ± 0.41 a
	530	19,464.10 ± 2433.39 b	19,934.36 ± 2825.05 b	10.48 ± 0.18 b
Bacteria	850	20,622.13 ± 2561.67 b	20,987.01 ± 2412.87 b	10.35 ± 0.10 b
	1100	20,167.14 ± 1693.96 b	20,838.70 ± 1589.23 b	10.65 ± 0.07 b
	1400	18,738.03 ± 2259.18 b	18,915.81 ± 2072.57 b	10.52 ± 0.43 b

**Table 3:** Interpretation ranking of the influence of soil physical and chemical properties on soil fungal community.

Environmental Factor	Explanatory Volume of Environmental Factors (%)	Pseudo-F	*p*
Sand	19.600	3.200	0.028
Clay	17.300	3.300	0.012
AK	15.900	3.700	0.016
pH	4.700	1.100	0.394
N/P	2.300	0.500	0.746
TN	1.700	0.400	0.860

**Table 4:** Interpretation ranking of the influence of soil physical and chemical properties on the soil bacterial community structure.

Environmental Factor	Explanatory Volume of Environmental Factors (%)	Pseudo-F	*p*
Clay	37.300	7.700	0.008
pH	17.100	4.500	0.008
AK	10.300	3.200	0.010
N/P	6.400	2.500	0.052
TN	4.800	1.600	0.184
C/P	3.900	1.300	0.238
Sand	2.800	1.100	0.342
C/N	1.300	0.500	0.746

## Data Availability

The data presented in this study are available upon request from the authors.

## References

[1] Yang C., Zhang X., Ni H., Gai X., Huang Z., Du X., Zhong Z. (2021). Soil Carbon and Associated Bacterial Community Shifts Driven by Fine Root Traits along a Chronosequence of Moso Bamboo (*Phyllostachys edulis*) Plantations in Subtropical China. Sci. Total Environ..

[2] Li Z.K., Zhang Y.R., Deng Z.W., Liu J.Y., Rong J.D., Chen L.G., He T.Y., Zheng Y.S. (2024). Effects of enclosure term on fine root functional traits of Moso bamboo (*Phyllostachys edulis*) in the Wuyi Mountains. Acta Ecol. Sin..

[3] Ye L.Q., Ku W.P., Liu J., Xu m.Y., Meng F.R., Fu W.J., Liu J., Jin J., Wu J.S. (2020). Effects of enclosure term on community structure and undergrowth diversity of (*Phyllostachys edulis*). Acta Ecol. Sin..

[4] Peng H., Chen H.W., Li Q.J., Shen Q.H., Zhou H.M. (2024). The effects of *Phyllostachys edulis* invasion in cunninghamia lanceolata forest on the diversity of fungal community. Soil Fertil. Sci. China.

[5] Wang X., Wang Y.X., Ji H.X., Shi M., Wang H.L., Song X.Z., Li Q. (2024). Response of phosphorus fractions in rhizosphere soil of Moso bamboo to nitrogen and biochar additions. Chin. J. Ecol..

[6] FAO (2010). Global Forest Resources Assessment 2010: Main Report.

[7] SFAPRC (2015). Forest Resources in China—The 8th National Forest Inventory.

[8] Xiang J., Gu J., Wang G., Bol R., Yao L., Fang Y., Zhang H. (2024). Soil pH Controls the Structure and Diversity of Bacterial Communities along Elevational Gradients on Huangshan, China. Eur. J. Soil Biol..

[9] Liu W., Guo S., Zhang H., Chen Y., Shao Y., Yuan Z. (2024). Effect of Altitude Gradients on the Spatial Distribution Mechanism of Soil Bacteria in Temperate Deciduous Broad-Leaved Forests. Microorganisms.

[10] Li Z., Wang Z., Zhang W., Zhu J., Chen B., Jiang L., Xu D., Li W., Liu J., He Z. (2024). Soil Environments Regulate Dominant Soil Fungal Communities along an Elevational Gradient in Subtropical Forests. Forests.

[11] Li H., Wang X.G., Liang C. (2015). Aboveground-belowground biodiversity linkages differ in early and late successional temperate forests. Sci. Rep..

[12] Song X.C., Guo L.M., Tian H.D., Deng X.j., Zhao L.S., Cao J.Z. (2017). Variation of soil microbial community diversity along an elevational gradient in the Mao’er Mountain forest. Acta Ecol. Sin..

[13] Li X., Lyu M., Zhang Q., Feng J., Liu X., Zhu B., Wang X., Yang Y., Xie J. (2024). Warming Reduces Priming Effect of Soil Organic Carbon Decomposition Along a Subtropical Elevation Gradient. Glob. Biogeochem. Cycles.

[14] Li Y., Feng X.X., Zhao F.Z., Guo Y.X., Wang L., Ren C.J. (2021). Structure characteristics of soil microbial community in *Quercus aliena* var. *acuteserrata* Forests at different altitudes in Qinling Mountains. Sci. Silvae Sin..

[15] Liu B.R. (2021). Recent Advances in altitudinal distribution patterns of biodiversity. Ecol. Environ. Sci..

[16] Xiong X.L., Ren Y.B., Lyu M.K., Li X.J., Nie Y.Y., Xie J.S. (2022). Distribution characteristics of soil organic carbon and total nitrogen storage in typical forest soils at different altitudes in Wuyishan Mountain. Res. Soil Water Conserv..

[17] Ma J.P., Pang D.B., Chen L., Wan H.Y., Chen G.L., Li X.B. (2022). Characteristics of soil microbial community structure under vegetation at different altitudes in Helan Mountains. Acta Ecol. Sin..

[18] Bryant J.A., Lamanna C., Morlon H., Green L.J. (2008). Microbes on mountainsides: Contrasting elevational patterns of bacterial and plant diversity. Proc. Natl. Acad. Sci. USA.

[19] Singh D., Takahashi K., Kim M., Chun J., Adams J.M. (2012). A Hump-Backed Trend in Bacterial Diversity with Elevation on Mount Fuji, Japan. Microb. Ecol..

[20] Zhang J., Guo Q., Sun Y.M., Lin C., Cai L.P., Zhang H.X. (2022). Ecological stoichiometric characteristics of soil carbon, nitrogen and phosphorus in Moso bamboo forests at different altitudes in Wuyi Mountain. J. Fujian Agric. For. Univ. (Nat. Sci. Ed.).

[21] Sun Y., Chen X., Zhong A., Guo S., Zhang H. (2023). Variations in Microbial Residue and Its Contribution to SOC between Organic and Mineral Soil Layers along an Altitude Gradient in the Wuyi Mountains. Forests.

[22] Zhang H.X., Lin C., Cheng H., Jin C.S., Xu Z.k., Wei Z.C., Ma X.Q. (2019). Variation of soil organic carbon content of Moso Bamboo forest along altitudinal gradient in Wuyi Mountain in China. Soils.

[23] Bao S.D. (2001). Soil Agrochemical Analysis.

[24] Guo M., Wu F., Hao G., Qi Q., Li R., Li N., Wei L., Chai T. (2017). Bacillus Subtilis Improves Immunity and Disease Resistance in Rabbits. Front. Immunol..

[25] Toju H., Tanabe A.S., Yamamoto S., Sato H. (2012). High-Coverage ITS Primers for the DNA-Based Identification of Ascomycetes and Basidiomycetes in Environmental Samples. PLoS ONE.

[26] Xiong R., Qian D., Qiu Z., Hou Y., Li Q., Shen W. (2024). Land-Use Intensification Exerts a Greater Influence on Soil Microbial Communities than Seasonal Variations in the Taihu Lake Region, China. Sci. Total Environ..

[27] Bai Y., Wei H., Ming A., Shu W., Shen W. (2023). Tree Species Mixing Begets Admixture of Soil Microbial Communities: Variations along Bulk Soil, Rhizosphere Soil and Root Tissue. Geoderma.

[28] Yan F., Zhao X., Zhao Y.P., Wang X.Y., Zhang Y.T., Liang Y.B., Wen Y.X., Chen Y.H. (2024). Effects of Mine Remediation Mode on Microbial Community Structure and Function in the Rhizosphere Soil of Robinia Pseudoacacia. Environ. Sci..

[29] Li Y., Yang H.V., Yu Q.L., Guo D.G., Zhang Q.X. (2024). Soil Microbial Diversity and Its Response to Heavy Metals in Yuncheng Salt Lake Wetland. Environ. Sci..

[30] Rao D.A., Liu P.Y., Zou L.Y., Teng Y., Yu H.Y., Yin G.T. (2022). Effects of long-term continuous cropping and reductive soil disinfestation on fungal community in flue-cured tobacco rhizosphere. Soil Fertil. Sci. China.

[31] Zhao Q.Q., Xie J.K., Gao Y.C., Zhang W., Wang J.N., Chen G.H. (2022). The distribution pattern of soil fungal community in coastal wetlands with different hydrologic conditions in the Yellow River Estuary. Acta Sci. Circumstantiae.

[32] Li Y.Y., Xu T.T., Ai Z., Ma F. (2021). Fungal community diversity and driving factors in rhizosphere soil of Caragana species across semi-arid regions. Chin. J. Appl. Ecol..

[33] Zheng Q., Hu Y., Zhang S., Noll L., Böckle T., Dietrich M., Herbold C.W., Eichorst S.A., Woebken D., Richter A. (2019). Soil Multifunctionality Is Affected by the Soil Environment and by Microbial Community Composition and Diversity. Soil Biol. Biochem..

[34] Yang X.J., Meng Z.Y., Li Y., Yang Y.Z., Li Y.G., Su Y., Li M. (2024). Analysis of fungal community structure in rhizosphere soil of dominant tree species in Helan Mountain. For. Eng..

[35] Zhang X.F., Zang C.P., Dong Q.M., Huo L.A., Tong Y.S., Zhang X., Yang Z.Z., Yu Y., Cao Q. (2024). Soil fungal community structure in perennial cultivated grassland around Qinghai Lake. Acta Agrestia Sin..

[36] Egidi E., Delgado-Baquerizo M., Plett J.M. (2019). A few Ascomycota taxa dominate soil fungal communities world wide. Nat. Commun..

[37] Wijayawardene N., Hyde K.D., Dai D.Q. (2018). Outline of Ascomycota. Fungal Divers..

[38] Zhang E.H., Liu P.P., He P., Jian Y., Xu Y.T., Chen C.X., Lu Y.Z., Lan X.Z., Suo L.S.M. (2024). Physiochemical properties and microbial community structure in rhizosphere soil of dracocephalum tanguticum. J. Agric. Sci. Technol..

[39] Huang F., Wang W.R., Rao X., He R., Wang J., Jian S.G., Shen W.j., Ren H. (2019). Soil improvements and microbial community development following establishment of plant communities in a tropical coral island. Acta Ecol. Sin..

[40] Zhao X., Liu H.L., Yang P., Qu Y.P., Wang S.M., Zhang X. (2019). Effects of drip irrigation on bacterial diversity and community structure in rhizosphere soil of alfalfa. Microbiol. China.

[41] Choudhary M., Sharma P.C., Jat H.S., Dash A., Rajashekar B., McDonald A.J., Jat M.L. (2018). Soil Bacterial Diversity under Conservation Agriculture-Based Cereal Systems in Indo-Gangetic Plains. Biotech.

[42] An X.R., Jiang S.T., Xie X.Y., Xu Y.C., Dong C.X., Shen Q.R. (2022). Effects of reducing chemical fertilizers combined with organic fertilizers on soil microbial community in litchi orchards. Chin. J. Appl. Ecol..

[43] Zhou Y.I., Jia X., Zhao Y.H., Wang Q., Ye X., An Y.R. (2021). Review on soil microbial patterns along the elevation gradient based on the knowledge mapping analysis. J. Ecol. Rural Environ..

[44] Klimek B., Jaźwa M., Choczyński M., Stolarczyk M., Niklińska M. (2020). The Drivers of Soil Microbial Communities Structure on Forest Stands along the Altitudinal Gradient in Western Carpathians. Acta Oecologica.

[45] Zhou Y.J., Jia X., Zhao Y.H., Chen N.N., Yan J., Tang J.Q., Wang Q., Liu L. (2021). Altitude distribution of fungal community in Huoditang in Qinling Mountains, Northwest China. Chin. J. Appl. Ecol..

[46] Peay K.G., Baraloto C. (2013). Fine Paul VA. Strong coupling of plant and fungal community structure across western Amazonian rainforests. ISME J..

[47] Liu D., Liu G.H., Chen L., Wang J.T., Zhang L.M. (2018). Soil pH determines fungal diversity along an elevation gradient in southwestern China. Sci. China Life Sci..

[48] Man B.Y., Xiang X., Luo Y., Mao X.T., Zhang C., Sun B.H., Wang X. (2021). Characteristics and influencing factors of soil fungal community of typical vegetation types in Mount Huangshan, East China. Mycosystema.

[49] Si G.C., Yuan Y.L., Wang J., Xia Y.Q., Lei T.Z., Zhang G.X. (2014). Microbial community and soil enzyme activities along an altitudinal gradient in Sejila mountains. Microbiol. China.

[50] Yu Y.Y., Li M.S., Liu X.L., Yin W.P., Li G.F., Mu L.Q., Cui X.Y., Cheng Z.C. (2020). Soil bacterial community composition and diversity of typical permafrost in Greater Khingan Mountains. Microbiol. China.

[51] Rousk J., Bååth E., Brookes P.C., Lauber C.L., Lozupone C., Caporaso J.G., Knight R., Fierer N. (2010). Soil Bacterial and Fungal Communities across a pH Gradient in an Arable Soil. ISME J..

[52] Zhao X.Y., Ju Z.J., Chen H., Fu Y., Zhao B., Zhang J.Y., Lu M.Q., Cui J.S., Zhang L.L. (2022). Spatial distribution of Quinolone Antibiotics and its correlation relationship with microbial community in soil of shijiazhuang city. Environ. Sci..

[53] Wang S.K., Zhao X.Y., Jia K.F., Gao B.L., Qu H., Mo W., Lian J., Chen M., Zhu Y.C. (2016). Soil bacterial diversity and its vertical distribution in Stipa klemenzii Community of Urad Desert Steppe. J. Desert Res..

[54] Liu B.R., Niu S.F., Zhang W.W. (2019). Effects of sol particle size on enzyme activities and the amount of soil microorganism in rhizosphere of *Caragana korshinskii* in desert steppe. Acta Ecol. Sin..

[55] Wang X.J., Zhang Z.C., Yu Z.Q., Shen G.F., Cheng H.F., Tao S. (2020). Composition and diversity of soil microbial communities in the alpine wetland and alpine forest ecosystems on the Tibetan plateau. Sci. Total Environ..

[56] Sun B.J., Zhang X.P., Jia S.X. (2013). The effect of soil physical and chemical properties on soil microbial community in agro-ecosystem. Soils Crops.

